# Abordando a Relação Clínica e a Comunicação de Notícias Difíceis com o Auxílio das Artes e dos Relatos Vivos: Addressing the Clinical Relationship and Communicating Difficult News with the Aid of the Arts and Living Stories

**DOI:** 10.1590/1981-52712015v43n4rb20190098

**Published:** 2019-10-14

**Authors:** Natiele Dutra Gomes Gularte, Maria Teresa Aquino de Campos Velho, Kelly Carvalho Silveira Gonçalves, Nagele Fatica Beschoren

**Affiliations:** I Universidade Federal de Santa Maria, Santa Maria, Rio Grande do Sul, Brasil.

**Keywords:** Medical Education., Empathy., Humanism., Educação Médica., Empatia., Humanismo.

## Abstract

**Introduction:**

Clinical Relationship (CR) is a more comprehensive term than doctor-patient relationship and is currently used to describe the health team’s contact with the patient and their families, as well as the team’s interaction with each other. To establish satisfactory interpersonal relationships, it is important to bring together communication and empathy skills that play a key role in building the CR. However, such topics are given little attention during medical training, with a more focused study on diseases and treatments. Rigid protocols and guidelines have accounted for a large proportion of undergraduate medical curricula, leaving less time for discussion of more abstract subjects related to the humanities and the subjectivity of patients, a view that is part of people’s reality.

**Objectives:**

To provide spaces and theoretical references for the discussion of topics less addressed in the medical curriculum and to encourage the importance of communication and empathy in health/disease processes.

**Methods:**

In view of the importance of providing medical students with humanizing experiences, we have proposed theoretical-practical workshops and conducted a qualitative research in order to evaluate the students’ views on learning about CR themes. In total, 33 second-year medical students participated in the project, by voluntary adhesion in response to an invitation distributed via electronic mail. Six theoretical-practical workshops were offered and executed in small groups and addressing topics such as: empathy, mourning, palliative care, end of life and communication of difficult news. The data collection methods were two focus groups conducted at the end of the six meetings encounters and participant observation, the notes from which were taken in a field diary.

**Results:**

Content analysis (Bardin, 2011) was the method chosen to study the results, following transcription and analysis of the speech of the groups. At the end of the analysis, four main categories (and five subcategories) emerged, of which two are highlighted in this article: “Art, cinema and literature evoke feelings: seeking humanism” and “Difficult news in the view of patients – the living experiences”. To this end, we used active methodologies such as small group work, use of videos and excerpts from films, music, reports, reading texts and conversation wheels.

**Conclusion:**

The participants’ statements showed satisfaction with the proposed theme and methodologies. We therefore evaluated the use of such techniques to sensitize students, leading them to reflect on the importance of empathy and communication in the work of physicians and health staff, developing interpersonal skills.

## RESUMO

**Introdução:**

A Relação Clínica (RC) é um termo mais abrangente do que relação médico-paciente e, atualmente, tem sido utilizado para descrever o contato da equipe de saúde com o paciente e seus familiares, além da interação da equipe entre si. Para estabelecer relações interpessoais satisfatórias, é importante reunir habilidades de comunicação e empatia, que têm um papel-chave na RC. No entanto, tais temas acabam sendo pouco discutidos durante a formação médica, em detrimento de um estudo mais focado em doenças e tratamentos. Rígidos protocolos e diretrizes têm tomado grande espaço dos currículos de graduação médica, deixando menos tempo para a discussão de temas mais abstratos, relacionados às humanidades e à subjetividade dos pacientes, visão que faz parte da realidade das pessoas.

**Objetivos:**

Propiciar espaços e referenciais teóricos para a discussão de temas menos abordados no currículo médico e incentivar a importância da comunicação e empatia nos processos de saúde/doença.

**Métodos:**

Diante da importância de proporcionar aos alunos de Medicina vivências humanizadoras, propusemos a realização de oficinas teórico-práticas e conduzimos uma pesquisa qualitativa com o intuito de avaliar a percepção dos estudantes sobre o ensino-aprendizagem de temas do escopo da RC. Ao todo, participaram do projeto 33 alunos a partir do quarto semestre do curso de Medicina, por adesão voluntária, após convite distribuído via correio eletrônico. Foram ofertadas seis oficinas teórico-práticas, executadas em um trabalho com grupos pequenos e abordando temas como: empatia, luto, cuidados paliativos, final de vida e comunicação de notícias difíceis. As técnicas para coleta dos dados foram a realização de dois grupos focais ao final dos seis encontros e a observação participante, cujos apontamentos eram tomados num diário de campo.

**Resultados:**

A análise de conteúdo (Bardin, 2011) foi o método escolhido para estudo dos resultados, após transcritas e analisadas as falas dos grupos. No final da análise, emergiram quatro categorias principais (e cinco subcategorias), das quais destacamos duas que serão discutidas neste artigo: “A arte, o cinema e a literatura evocam os sentimentos: buscando o humanismo” e “As notícias difíceis na visão dos pacientes – as experiências vivas”. Para isso, foram utilizadas metodologias ativas, como trabalho em pequenos grupos, uso de vídeos e trechos de filmes, músicas, relatos, leitura de textos e rodas de conversas.

**Conclusão:**

Os depoimentos dos participantes demonstraram satisfação com as temáticas e metodologias propostas. Dessa forma, analisamos o auxílio de tais técnicas para sensibilizar os alunos, levando-os a refletir sobre a importância da empatia e da comunicação no atuar dos médicos e equipe de saúde, desenvolvendo habilidades de relação interpessoal.

## INTRODUÇÃO

Uma relação interpessoal adequada é uma ferramenta imprescindível ao trabalho médico. Para isto, é preciso mesclar habilidades de comunicação e empatia, favorecendo a troca de saberes e informações com pacientes, familiares e equipe de saúde. A empatia é descrita como a capacidade de perceber, considerar e entender os sentimentos do outro, sem necessariamente ter experimentado situações semelhantes^1^. Essa característica tem sido muito discutida na educação médica atual, pois a formação de alunos que tenham mais empatia pode se refletir numa Relação Clínica (RC) de maior qualidade. Para Halpern^2^, a empatia aproxima o médico dos pacientes, facilitando as trocas necessárias a um bom diagnóstico e tratamento. Outros autores apontam que essa característica pode ser moldada ao longo da graduação e pesquisas afirmam que ela pode, inclusive, diminuir com o avançar dos semestres do curso^1,3,4^.

Quanto às habilidades de comunicação, saber o que falar e como falar é um atributo essencial do médico, que precisa transmitir ao paciente o diagnóstico, os métodos que serão empregados para confirmá-lo, as orientações quanto ao tratamento, pós-tratamento e, inclusive, as notícias difíceis. Salienta-se que o domínio dos últimos consensos e diretrizes, dos tratamentos mais modernos e da tecnologia faz parte do exercício da profissão médica. Mas tão importante quanto isso é o uso adequado de tempo pertinente na consulta e a informação clara e compreensível a respeito das doenças e enfrentamentos que o paciente necessitará fazer. Uma RC sólida está diretamente relacionada à aderência terapêutica, pois, como já dizia Balint^5^, a personalidade do médico é o primeiro medicamento que se administra ao paciente. Quanto maior o treinamento nesses aspectos de relacionamento interpessoal, mais fácil essa tarefa se torna^6^.

As diretrizes curriculares do curso médico já têm previsto uma formação mais voltada aos elementos subjetivos da RC, equilibrando o aprendizado técnico-científico com o aprendizado de humanidades e sentimentos^7^. Apesar disso, empiricamente notamos a pouca valorização da temática entre os estudantes e, inclusive, entre muitos professores da escola médica.

As metodologias ativas têm sido uma opção para a abordagem de temas das humanidades no ensino em geral. Gracia^8^ destaca que tocamos as pessoas, entre elas os alunos de Medicina, sensibilizando-as e evocando sentimentos humanitários e solidários que podem ser despertados e fortificados por meio da arte, do cinema, da leitura habitual e de clássicos. Essas metodologias têm o intuito de estimular a participação ativa do aluno na busca pelo conhecimento, evocando a reflexão, por meio da utilização de práticas variadas, evitando apenas a transmissão do conhecimento do professor para o aluno de forma passiva^9^.

Assim, tendo em vista a importância da RC na prática médica e a ampla gama de práticas disponíveis para sua abordagem, torna-se necessário propor novas formas de inserir os alunos de Medicina em um contexto de ensino-aprendizagem mais ativo e humanizado, quando possível, nas disciplinas cabíveis. Destacamos a relevância de propiciar espaços para discussão de temas por vezes considerados tabus, porém constantemente vivenciados durante a prática profissional. A dificuldade frequente em abordar assuntos como o enfrentamento do luto e da morte, a empatia frente ao sofrimento do paciente e a comunicação de notícias difíceis talvez advenha de uma história curricular que preteriu essas nuances por anos. Antes das reformas curriculares, os alunos de Medicina eram expostos primeiramente ao contato com o corpo sem história, nas aulas de anatomia. Apenas após alguns semestres enfrentavam os dilemas do contato com os pacientes reais, seus sentimentos, emoções, questionamentos e ideias^10^.

No entanto, ao alvorecer do século XXI, emergiu outro paradigma não paternalista. Por uma série de mudanças culturais, deparamo-nos com uma autonomia maior do paciente e o incentivo a essa condição. Devido à importância de respeitar esse princípio bioético, tornou-se relevante valorizar a pessoa, o doente em sua subjetividade e contexto. Dessa forma, a RC e o bom vínculo entre médico, paciente e família destacam-se como formas de auxiliar a redução das demandas por má prática médica, tão comuns atualmente^11^.

Diante do exposto, fica evidente a relevância do debate dessas questões na graduação médica, visando formar alunos mais reflexivos e sensíveis ao sentimento alheio. Os atores envolvidos na formação acadêmica atual precisam buscar alternativas de ensino para evitar que os anseios por salvar e ajudar, tão presentes nos alunos dos semestres iniciais, não se dissolvam ao longo da pesada rotina de estudos e estágios. Assim, a ideia das oficinas do projeto era propiciar espaços para exposição de sentimentos e emoções sobre os assuntos tratados, avaliando a percepção dos alunos após essas novas experiências didáticas.

## MÉTODO

Este estudo de intervenção, descritivo, de abordagem qualitativa com técnica de grupo focal, deriva de uma dissertação de mestrado, intitulada “A Relação Clínica e o processo de comunicação e informação na formação médica: resgate e proposta”. O projeto inicial foi aprovado pelo Comitê de Ética e Pesquisa da Universidade Federal de Santa Maria (CEP/UFSM CAAE 69059317.1.0000.5346). Neste artigo, apresentaremos duas das categorias elencadas na dissertação, que contém resultados mais amplos.

Participaram deste estudo 33 alunos de Medicina da Universidade Federal de Santa Maria (UFSM) que cursavam do quarto semestre em diante. Tal escolha se fez porque a partir da Semiologia, disciplina ministrada no quarto semestre, os alunos intensificam seu contato com o paciente. A UFSM é uma universidade pública, localizada na região central no Rio Grande do Sul, e seu hospital-escola é o Hospital Universitário de Santa Maria (HUSM), que conta com 356 leitos e atendimento 100% pelo Sistema Único de Saúde. O curso de Medicina foi criado em 1954 e atualmente oferece 108 vagas anuais para ingresso.

Num primeiro momento, para concretizar o estudo, foram ofertadas, como projeto de extensão, seis oficinas teórico-práticas sobre temas atinentes à RC, tais como comunicação de notícias difíceis, luto e cuidados de final de vida, empatia e habilidades de comunicação. O projeto ofereceu um número limitado de vagas, considerando a importância de trabalhar esses assuntos em grupos pequenos. Todos os 33 alunos inscritos voluntariamente foram admitidos, e as oficinas, com duração de duas horas, ocorreram entre os meses de outubro e novembro de 2017. Foram realizadas atividades como estudo e leitura de casos ético-clínicos, apresentação e discussão de trechos de filmes, dramatizações, procura por artigos científicos relacionados ao tema, reportagens, relato de experiências vividas e problemas enfrentados pelos alunos em seu cotidiano como entrevistadores.

No primeiro encontro foi explicada toda a dinâmica que seria seguida no projeto e que, no final, o grupo seria convidado a participar de dois grupos focais com o intuito de avaliar suas percepções sobre a abordagem desses temas na graduação e no projeto. Do total, 15 alunos participaram voluntariamente dos grupos focais.

### Coleta de dados nos grupos focais e análise dos dados

A técnica dos grupos focais foi escolhida porque proporciona maior interação e troca de experiências entre os participantes, pois as respostas podem ser complementadas ou questionadas por eles, permitindo o surgimento de descobertas e composições não previstas pelos pesquisadores^12^. Os alunos foram dispostos em círculos e convidados a debater sobre os temas com base em perguntas norteadoras lançadas pelas pesquisadoras. As falas foram gravadas e transcritas de forma literal pela principal condutora do grupo focal no dia posterior ao encontro. Após, foi realizada a leitura flutuante do material com análise do conteúdo e categorização. As pesquisadoras elegeram, separadamente, temáticas emergentes das falas dos alunos de acordo com a repetição e pertinência dos conteúdos – metodologia proposta por Bardin^13^. Uma vez propostas as categorias, de maneira individual, elas foram confrontadas e debatidas pelas pesquisadoras, sendo admitidas quatro categorias principais, com cinco subcategorias.

Optou-se por abordar duas delas neste artigo: “A arte, o cinema e a literatura evocam os sentimentos: buscando o humanismo” e “As notícias difíceis na visão dos pacientes – as experiências vivas”. Com o intuito de manter a confidencialidade dos dados, os alunos foram identificados pela letra A, seguida por algarismos arábicos de um até 15. As idades dos participantes variaram entre 20 e 37 anos, e todos assinaram o Termo de Consentimento Livre e Esclarecido (TCLE) antes de iniciarem a participação no projeto.

## RESULTADOS E DISCUSSÃO

As duas categorias apresentadas a seguir foram eleitas para serem descritas e comentadas neste artigo, considerando a importância dos temas no ensino da RC por meio do emprego de metodologias ativas. O uso das artes como estratégia de humanização há muito já vem sendo adotado. Algo que pode ser considerado inovador e ainda pouco descrito foi o convite feito a pacientes reais que compartilharam seus relatos e vivências acerca do seu processo de adoecimento e a comunicação das notícias difíceis pelos profissionais da saúde. Percebeu-se o impacto ocasionado às pessoas que confidenciaram seus relatos e ao grupo de alunos participante do projeto pela escuta das experiências reais e rememoradas nos encontros. Muitas vezes, tal situação é mimetizada pela utilização de atores que encenam problemas de saúde. Porém, o emprego de situações reais pode facilitar o desenvolvimento da empatia e compaixão pelo contexto do outro.

As falas dos alunos foram transcritas de maneira literal após a realização dos grupos focais e serviram de base para discussão acerca de seus conteúdos. A seguir, serão descritos os achados referentes às categorias eleitas para o presente artigo. Destaca-se que a apresentação dos resultados, sua análise e discussão serão realizadas conjuntamente.

### A arte, o cinema e a literatura evocam os sentimentos: buscando o humanismo

Essa categoria engloba a utilização da música, da leitura, da poesia, das artes, dos filmes e dos vídeos como técnicas para sensibilizar e humanizar os alunos e, dessa forma, trabalhar o ensino médico não apenas com aulas teóricas formais. Nem todas as disciplinas e os conteúdos do currículo podem ser trabalhados dessa forma, mas cabe ressaltar que os docentes precisam estar empenhados em identificar situações que podem e devem ser trabalhadas com metodologias diferentes, buscando um viés mais humanístico. Sabe-se que questões mais subjetivas que envolvam a ética, a empatia, a comunicação, entre outras temáticas, podem ser mobilizadas nas pessoas por meio da utilização desses recursos^11^. A preocupação em buscar maior humanização para auxiliar o estudante de Medicina tem sido descrita por vários autores^14,15,16^. No entanto, pouco se tem avaliado a eficácia dessas estratégias e a percepção dos alunos acerca delas^11^. Livros e artigos podem auxiliar na obtenção de capacidades técnicas, porém, para formar alunos mais reflexivos, ativos nessas questões e aptos a considerarem a subjetividade do paciente, pode-se lançar mão de metodologias ativas, como materiais audiovisuais, para promover a reflexão crítica e a introspecção que tais assuntos requerem.

Quando perguntados acerca de sua opinião sobre as metodologias utilizadas no projeto, os alunos discutiram e elaboraram as falas descritas a seguir:

*[…] eu gostei dos encontros porque […] eram relatos, eram conversas, pedaços de filmes, leituras, a gente foi aprendendo com exemplos, é bem diferente daquela aula teórica […]. Esses exemplos acho que ajudaram muito, me fizeram pensar muito mais do que seu eu tivesse tido uma aula, como as que a gente tem.* (A3)

*[…] um aspecto positivo foram os filmes que traziam a história de algum acontecimento, alguma notícia que tinha que dar. Por exemplo, o médico que tinha câncer e como ele ia receber ou como as pessoas falaram isso pra ele. Então, eu acho que nessa parte ajudou, porque a gente conseguiu ver exemplos práticos, não ficou só na teoria. Foi através do filme, mas ajudou a gente a ver como acontece isso de dar a notícia ou como é que as pessoas recebem também.* (A2)

*[…] eu achei a parte didática bem legal mesmo. Me atraiu, pelo menos, por ser essa questão da gente falar, de ver um vídeo. Até eu fui procurar os vídeos em casa. Os diálogos também acho que o fato da gente não ter que levar pra casa pra ler, de ser ali, faz com que a gente preste atenção, porque senão, eu, pelo menos, acabo deixando para outra hora e não faço. Achei os temas bem abordados, temas bons.* (A1)

Destacamos a dificuldade em abordar temas mais subjetivos, como as humanidades e a relação médico-paciente, de maneira não apenas teórica, mas que aguçasse a sensibilidade e se mostrasse prática e concretizável. Alguns currículos de graduação médica ainda abordam esses assuntos em aulas teóricas com turmas grandes, o que dificulta a expressão de afetos e sentimentos por parte dos alunos^9^, informação que também foi evidenciada no presente estudo. Os alunos enfatizaram a importância de grupos menores para a discussão e a autoexpressão frente a determinados temas e aulas. Relataram a dificuldade dessa ação em grupos maiores, por medo de exposição, constrangimentos e pressa, entre outras circunstâncias. Podemos verificar essa afirmação nas expressões adiante:

*[…] na nossa turma de 60 alunos, na aula de notícias difíceis, não teve uma pergunta pra professora, mas não foi porque não tinha dúvidas, foi porque ou estavam com vergonha de perguntar ou estavam com medo de perguntar e atrasar o colega que queria ir embora […] no grupo menor fica muito mais fácil falar.* (A15)

*[…] porque, se a gente estivesse numa sala que tivesse mais gente, o professor prefere que ninguém fale nada, para poder acabar logo a situação e ir em frente […] mas pelo menos para a gente, é muito melhor [grupos pequenos], para se sentir à vontade para falar. Eu particularmente acho que falei até bastante no grupo.* (A4)

*Porque se falar do mesmo assunto numa sala com 60 pessoas, alguns não querem nem saber disso, não querem ouvir, é muito difícil. Não vai fluir.* (A6)

Segundo Blasco (p. 12)^16^, para tratar de temas que envolvam mais sentimentos e menos teoria, a exemplo dos cuidados paliativos, do luto, das notícias difíceis, “é necessário discutir significados ao invés de apenas estabelecer protocolos”. Assim sendo, nota-se pouco espaço para a discussão e aprendizado de como lidar com as emoções despertadas nos alunos ao se depararem com o sofrimento dos pacientes. O uso das artes, como proposto nas oficinas do projeto, pode auxiliar sobremaneira nessas questões^17–20^. Os fragmentos das falas dos alunos descritos a seguir ilustram essa questão:

*Eu quis participar do projeto por essa deficiência no curso mesmo. Acho que o nosso curso está bastante voltado para a técnica e eu acho que acaba faltando um pouco essa humanização da medicina. Eu acho que aqui a gente pôde discutir, conversar, cada um pôde falar […] não era aquela coisa maçante de* datashow *ali, professor falando e tu ouvindo quietinho.* (A6)

*Eu acho que o que eu mais gostei, o que me interessou para entrar no projeto foi essa deficiência no curso que tem, como a gente disse, da disciplina de Relação Médico-Paciente que pra mim não acrescentou quase nada. E daí eu gostei dos encontros porque eram metodologias diferentes, a gente podia compartilhar o que via de certo ou errado no HUSM.* (A8)

De acordo com as teorias da neuroeducação, o cérebro precisa se emocionar para aprender^17^. Acreditamos que esta seja uma proposta para o ensino da RC. Diminuir o tempo de aulas teóricas e propiciar contato com cenas, relatos, músicas e filmes impactantes referentes aos assuntos que se deseja abordar. Cabe relembrar que determinados conteúdos não são tão fáceis de abordar nessa perspectiva, principalmente os mais densos e iniciais, como anatomia, genética e patologia, entre outros. No entanto, pôde-se perceber, com base nos relatos a seguir, o quanto as metodologias ativas, quando adequadamente empregadas, são capazes de despertar o interesse dos alunos que se colocam como protagonistas do seu processo de ensino-aprendizagem.

Nesse contexto, a literatura demonstra a importância de se modificar a postura na relação professor-conhecimento-aluno^18,21^. O foco deve ser desviado para o desenvolvimento de conhecimentos por meio do pensamento e da reflexão, evitando a simples reprodução de saberes transmitidos de geração em geração^18^. Percebemos que esse foi um ponto bastante frisado pelos alunos ao longo dos encontros: a insatisfação com a discussão de temas da RC em turmas grandes, com aulas predominantemente teóricas e expositivas. As falas a seguir demonstram a valorização dos alunos pelo fato de se sentirem mais inseridos no processo didático.

*[…] enquanto no projeto a gente não tinha essa obrigatoriedade [de ler os temas em casa], mesmo que falasse o mesmo assunto das nossas aulas, a gente ia mais leve, a gente ia querendo ouvir, querendo conversar. É bem diferente da aula, que a gente fica pensando quanto tempo falta para acabar.* (A4)

*Nas aulas de história de medicina, com o professor X, que é um cara tipo, ele pensa fora da caixa assim, de um médico. Ele tem mestrado e doutorado em educação, já começa assim. Então é diferente. E é isso aí, sabe, eu acho que foi o* ***único*** *(grifo nosso) momento que a gente teve durante a graduação pra refletir alguma coisa.* (A6)

O grupo inicial de 33 alunos foi dividido em dois menores, sendo que cada oficina foi realizada duas vezes, englobando acadêmicos dos diversos semestres do curso. Essa mescla de troca de saberes e experiências entre alunos de diferentes semestres foi um destaque positivo da nossa metodologia, bem como o trabalho em pequenos grupos. Cabe destacar que discutir emoções e expressar sentimentos, em conjunto, auxilia nas identificações/soluções de problemas cotidianos, diminui o sofrimento e pode aumentar a confiança e a capacidade de resiliência na prática clínica^22^.

Os relatos expostos até o momento corroboram o descrito na literatura sobre a importância de propor a utilização de vídeos, filmes ou literatura e demonstrar aos alunos como essas metodologias podem ser eficazes na construção de humanidades, conduzindo às atividades práticas e às discussões em grupo^15,19,20,22^. Isso parece ir ao encontro das expectativas dos alunos que anseiam por ampliar seus horizontes. Como exemplo, citamos que as discussões sobre luto, cuidados paliativos e notícias difíceis na visão dos pacientes foram solicitadas pelos próprios alunos ao longo do projeto.

### As notícias difíceis na visão dos pacientes – as experiências vivas

As notícias difíceis são definidas como qualquer comunicação que transforme, impacte ou redirecione negativamente os planos de vida de uma pessoa^23^. Diante da necessidade de conscientizar os alunos de que essa é uma tarefa médica e da importância de saber dar o suporte emocional necessário ao paciente que recebe uma notícia difícil, realizamos duas oficinas que abordaram essa temática. No final de um desses encontros, um aluno sugeriu que seria interessante abordar esse tema na perspectiva do paciente que recebe essa notícia. Essa ideia foi acatada pelas pesquisadoras, que providenciaram uma terceira oficina que englobou esse aspecto do processo de comunicação das notícias difíceis. Essa é uma das particularidades de algumas formas qualitativas de pesquisar e da própria metodologia ativa. É permitido convergir e aceitar propostas que são levantadas e consideradas importantes pelos participantes ao longo do projeto.

Buscaram-se, então, alguns profissionais e um estudante que experimentaram processos sérios e difíceis de adoecimento para relatarem ao grupo suas experiências de como foi a RC nessas situações e de como lhes foram comunicados os diagnósticos e prognósticos – as notícias difíceis. Os convidados foram um estudante da graduação que ficou paraplégico aos 18 anos de idade após acidente automobilístico; um médico que recebeu o diagnóstico de neoplasia cerebral no transcorrer da primeira gestação de sua esposa (também médica); e, por último, uma médica que havia sofrido um acidente automobilístico, permanecendo várias semanas internada em unidade de terapia intensiva. Percebemos que os relatos auxiliaram a despertar a empatia dos participantes e foram um dos destaques positivos na avaliação final do projeto, como percebido nas seguintes falas:

*Quando trouxeram lá o depoimento do Y. e do outro casal de médicos, também foi muito legal de ver, sabe. Como se fosse um caso clínico […], eles passaram por aquilo, eles falam com emoção sobre aquilo.* (A6)

*Acho que a [oficina] das notícias difíceis na visão dos pacientes foi a que eu mais gostei. Porque eu acho que eu me coloquei um pouco no lugar deles, aquele casal de médicos falando sobre como os médicos falaram com eles: “Ah… conta sua historinha triste” […].* (A1)

*Então eu acho que só escutar o relato da médica nos fez perceber a real importância disso, de se colocar na pele da pessoa para ver o que ela está passando e o que a gente pode fazer para melhorar ainda mais os nossos futuros atendimentos.* (A3)

*[…] a oficina que eu acho que voltei pra casa mais pensando foi a do relato da pediatra, de como a gente não se vê no lugar do outro e às vezes trata o paciente como uma atração.* (A9)

A literatura traz vários exemplos do treinamento de habilidades de comunicação e notícias difíceis por meio de simulações de consultas com pares ou pacientes simulados^22,24,25^, mas há pouco material sobre a abordagem desse tema com uso de relatos de pacientes reais. Além disso, o fato de os relatos serem de um estudante e de médicos que estavam na condição de pacientes parece ter despertado mais empatia e sensibilidade nos ouvintes. Os depoimentos trouxeram à tona a possibilidade de adoecimento dos próprios alunos, das dores físicas e psíquicas e da finitude humana em qualquer faixa etária, por vezes esquecida diante da profissão médica. Os alunos pontuaram que por vezes observavam e percebiam o adoecimento como algo distante de si, como evidenciado nas falas a seguir:

*Eu também, a oficina de que eu mais gostei foi a do relato da médica paciente […] eu nunca tinha tido contato com um paciente que me contasse com detalhes o que foi difícil passar, como foi tratado, sabe?* (A5)

*Pra mim foi o relato da médica, porque eu nunca tinha parado pra pensar, porque parece que a gente é imune a tudo. Então, se acontecesse com a gente, como seria o atendimento?* (A2)

*Eu acho que essa parte de trazer um relato, principalmente de uma pessoa que é da mesma área da gente, foi muito bom pra gente perceber como é que é a real empatia.* (A12)

*Eu acho que foi muito interessante porque acabou me sensibilizando mais quanto ao paciente. Principalmente o relato da doutora, o que aconteceu com ela, e que a gente um dia também vai ser paciente.* (A6)

As narrativas “vivas” têm o poder de narrar nossas humanidades e, por isso, adquirem caráter formativo, crítico e transformador^26,27,28^. A respeito desses aspectos, os participantes relataram a importância de uma RC bem estruturada, compromissada, compassiva e respeitosa, assim como verificaram que o modo de transmitir notícias ao paciente pode influenciar de forma muito positiva ou negativa quem as recebe. Sontag^29^ já referia que as fantasias que os pacientes fazem a respeito de suas doenças podem ser maiores que a doença em si. Todas as circunstâncias que envolvem o processo de adoecimento precisam ser esclarecidas, clarificadas e dimensionadas dentro da realidade para o auxílio à adaptação a tais processos. Para isto, é necessário que os profissionais de saúde estejam munidos de ferramentas que proporcionem melhor desempenho nos aspectos da RC. Na pesquisa, os participantes observaram, ainda, exemplos positivos e negativos extraídos dos relatos escutados:

*[…] o Y., que é a felicidade em pessoa [referindo-se ao aluno com paraplegia]. E ele conseguiu, ele teve um monte de profissionais que fizeram com que ele tivesse esse desfecho bom.* (A6)

*Eu nunca tinha parado pra pensar como eu ia dar assim uma notícia difícil. Mas vendo aqueles exemplos, eu vi como eu não quero ser, sabe?* (A9)

*E o bom é que a gente pôde ter um exemplo de quando a gente faz a coisa correta, que foi o exemplo do Y., como ele é bem resolvido com a situação dele […]*. (A5)

*[…] mas também a participação do Y. eu acho que foi muito importante, sobre as formas diferentes como a gente age diante do paciente e como isso impacta a vida dele não só no tratamento mas pra além do tratamento, sabe?* (A1)

*[…] então eu acho que mudou completamente a minha visão sobre como abordar o paciente […] e poder ter contato com essas histórias que a gente teve eu acho que mudou completamente a minha visão de perguntar como o paciente está sentindo, o que ele está pensando, pelo que ele está passando. Acho que ajudou bastante nisso.* (A15)

Esses relatos proporcionam reflexões para que os participantes se tornem conscientes de suas formas individuais de comprometimento. Turato^30^ complementa esse raciocínio, postulando que quem não possuir essa sensibilidade e disponibilidade interior de acolher as angústias e as ansiedades do outro não será um bom investigador clínico. Tampouco será um médico empático, condoído, que respeita e considera seus pacientes, em todos os âmbitos de contato com eles. O profissional precisa, igualmente, estar consciente de suas próprias limitações e ultrapassá-las quando possível, cabendo aos docentes ofertar o auxílio saudável e necessário para o desenvolvimento de habilidades de RC.

Por fim, a escuta de histórias reais pelo grupo de estudo foi capaz de sensibilizar e gerar interpretações, indo ao encontro dos objetivos definidos pelas pesquisadoras durante a organização do projeto. Essa experiência inovadora, ao colocar os alunos em contato com histórias de vidas impactantes, no que tange à comunicação médico-paciente, pode servir de exemplo para outras atividades no curso de Medicina, com o intuito de formar alunos mais reflexivos sobre as próprias práticas e que respeitem os valores, perspectivas e visões dos seus pacientes. Ainda, é salutar lembrar e adicionar um enfoque mais humano nas relações dos médicos com seus pacientes e entre as equipes de trabalho, o que por vezes é esquecido numa rotina corrida de atendimentos e procedimentos, onde os pacientes viram números, diagnósticos e leitos.

## CONSIDERAÇÕES FINAIS

O ensino médico atual necessita estimular o aluno a compreender que está diante de um paciente com uma história de vida, valores, desejos e crenças, e não apenas de um corpo doente. É preciso desenvolver sensibilidade, afeto e empatia para perceber que se está cuidando de um outro (paciente e/ou família) que sofre. Percebeu-se, durante a realização do trabalho, a pertinência da temática e o potencial de sensibilização por meio das metodologias propostas (relatos de experiências vividas, trechos de filmes e músicas, leitura e interpretação de situações), destacando-se como um dos pontos positivos a assiduidade dos participantes nas oficinas.

Reiteramos os aspectos discutidos ao longo do artigo, destacando a importância de abordar os valores e as questões da vida do paciente, não focando apenas os sinais e sintomas que serão conduzidos com exames diagnósticos e tratamentos quando necessário. Esse contato harmonioso entre médico--paciente-equipe-família pode transformar-se em instrumento fundamental para esclarecer os sintomas e orientar o tratamento das doenças. Uma comunicação eficaz também é essencial para se estabelecer um relacionamento respeitoso e honesto – componentes fundamentais do cuidado.

Acreditamos ter atingido o objetivo principal do projeto, de aliar o saber científico aos sentimentos, escuta e linguagem adequadas, além de mobilizar sensações e emoções no intuito de promover empatia. É importante continuar estimulando, na graduação, o resgate da medicina humanizada associada às evidências científicas. Os profissionais envolvidos no ensino médico podem trabalhar para mostrar que o saber e o sentir podem e devem caminhar juntos, visando maior satisfação profissional e habilidade na arte de cuidar e curar. Pois, como expresso no famoso aforismo de autor desconhecido, cabe ao médico “curar algumas vezes, aliviar frequentemente e consolar sempre”.

## References

[1] Hojat M, Mangione S, Nasca TJ, Rattner S, Erdmann JB, Gonnella JS, Magee M. An empirical study of decline in empathy in medical school. Med Educ. [online]. 2004. [capturado em: 25 mar. 2018]; 38(9): 934–941. Disponível em: https://onlinelibrary.wiley.com/doi/full/10.1111/j.13652929.2004.01911.x?sid=nlm%3Apubmed 15327674 10.1111/j.1365-2929.2004.01911.x

[2] Halpern J From Detached Concern to Empathy: Humanizing Medical Practice. New York: Oxford University Press, 2001.

[3] Hojat M, Maxwell K, Brainard G, Herrine SK, Isenberg GA, Veloski J, The devil is in the third year: a longitudinal study of erosion of empathy in medical school. Acad Med. [online]. 2009. [capturado em: 25 mar. 2018]; 84(9): 1182–91. Disponível em: https://journals.lww.com/academicmedicine/fulltext/2009/09000/The_Devil_is_in_the_Third_Year__A_Longitudinal.12.aspx#pdf-link 19707055 10.1097/ACM.0b013e3181b17e55

[4] Moreto G, Blasco PG, Pessini L, Benedetto MAC. La erosión de la empatía en estudiantes de Medicina: reporte de un estudio realizado en una universidad en São Paulo, Brasil. Aten Fam. [online]. 2014. [ capturado em: 10 abr. 2018]; 21(1): 16–19. Disponível em: https://www.sciencedirect.com/journal/atencion-familiar/vol/21/issue/1

[5] Balint M O médico seu paciente e a doença. São Paulo: Livraria Atheneu, 1988.

[6] Bonamigo EL, Destefani AS. A dramatização como estratégia de ensino da comunicação de más notícias ao paciente durante a graduação médica. RevBioet. [online]. 2010. [capturado em: 25 mar. 2018]; 18(3): 725–742. Disponível em: http://www.revistabioetica.cfm.org.br/index.php/revista_bioetica/article/viewFile/596/602

[7] Brasil. Ministério da Educação. Resolução n° 3, de 20 de junho de 2014. Institui Diretrizes Curriculares Nacionais do Curso de Graduação em Medicina e dá outras providências. Diário Oficial da União, Brasília, 23 de junho de 2014 – Seção 1, p. 8–11.

[8] Gracia D The mission of ethics teaching for the future. International Journal of Ethics Education. [online]. 2016. [capturado em: 15 mar. 2018]; 1(1): 7–13. Disponível em: https://link.springer.com/journal/40889/1/1

[9] Bacich L, Moran J.Metodologias ativas para uma educação inovadora:uma abordagem teórico-prática. Porto Alegre: Penso, 2018.

[10] Quintana AM A angústia na formação do estudante de medicina. RevBrasEduc Med. [online]. 2008. [capturado em: 16 mar.2018]; 32(1): 7–14. Disponível em: http://www.scielo.br/scielo.php?pid=S0100−−55022008000100002&script=sci_abstract&tlng=es

[11] Blasco PG. É possível humanizar a Medicina? Reflexões a propósito do uso do Cinema na Educação Médica. O Mundo da Saúde. [online]. 2010. [capturado em: 22 abr, 2018]; 34(3): 357–367. Disponível em: https://sobramfa.com.br/wp-content/uploads/2014/10/2010_dez_e_possivel_humanizar_a_medicina.pdf

[12] Ressel LB, Beck CLC, Gualda DMR, Hoffmann I, Silva RM, Sehnem GD. O uso do grupo focal em pesquisa qualitativa. Texto contexto - enferm. [online]. 2008. [capturado em: 15 abr. 2018]; 17(4): 779–786. Disponível em: http://www.scielo.br/scielo.php?script=sci_arttext&pid=S0104−−07072008000400021&lng=en. 10.1590/S0104-07072008000400021

[13] Bardin L Análise de Conteúdo. São Paulo: Edições 70, 2011.

[14] Ayres JRCM, Rios IC, Schraiber LB, Falcão MTC, Mota A. Humanidades como disciplina da graduação em Medicina. RevBrasEduc Med. [online]. 2013. [capturado em: 15 mar. 2018]; 37(3): 455–463. Disponível em: http://www.scielo.br/scielo.php?script=sci_arttext&pid=S0100−−55022013000300019&lng=en&nrm=iso

[15] Gallian M, Pondé LF, Ruiz R. Humanização, humanismos e humanidades: problematizando conceitos e práticas no contexto da saúde no Brasil. RevInt Hum Med. [online]. 2012. [capturado em: 23 jun. 2018]; 1(1): 5–15. Disponível em: https://journals.epistemopolis.org/index.php/hmedicas/article/view/1293/847

[16] Blasco PG. O humanismo médico: em busca de uma humanização sustentável da Medicina. RevBras Med. [online]. 2011. [capturado em: 12 abr. 2018]; 68 (esp oncologia): 4–12. Disponível em: https://sobramfa.com.br/wp-content/uploads/2014/10/2011_mai_o_humanismo_medico_humanizacao_sustentavel_da_medicina.pdf

[17] Mora F Neuroeducação: só é possível aprender aquilo que se ama. Espanha: Alianza Editorial, 2010.

[18] Carabetta V Junior Metodologia ativa na educação médica. RevMed São Paulo. [online]. 2016. [capturado em: 10 mai. 2018]; 95(3): 113–121. Disponível em: http://www.revistas.usp.br/revistadc/article/view/103675/120891

[19] Mortalla TD. Bioética y cine: de la narración a la deliberación. Madrid: San Pablo y Universidad Pontificia de Comillas, 2010.

[20] Gallian D A literatura como remedio: os clássicos e a saúde da alma. São Paulo: Martin Claret, 2017.

[21] Martin SK The consultation observed simulated clinical experience: training, assessment, and feedback for incoming interns on requesting consultations. Acad Med. [online]. 2018. [capturado em: jan. 2019]; 93(12): 1814–1820. Disponível em: https://journals.lww.com/academicmedicine/Abstract/2018/12000/The_Consultation_Observed_Simulated_Clinical.28.aspx 29923893 10.1097/ACM.0000000000002337 PMC6265083

[22] Instituto Nacional de Câncer. Comunicação de notícias difíceis: compartilhando desafios na atenção à saúde. [capturado em: 06 abr. 2018]. Rio de Janeiro; 2010. Disponível em: http://bvsms.saude.gov.br/bvs/publicacoes/comunicacao_noticias_dificeis.pdf

[23] Lino CA, Lustosa KA, Oliveira RAS, Feitosa LB, Caprara, A. Uso do protocolo Spikes no ensino de habilidades em transmissão de más notícias. RevBrasEduc Med. [online]. 2011. [capturado em: 17abr 2018]; 35(1): 52–57. Disponível em: <http://www.scielo.br/scielo.php?script=sci_arttext&pid=S0100-55022011000100008&lng=en&nrm=iso>. ISSN 0100–5502. 10.1590/S0100-55022011000100008

[24] Marco MAD, Vessoni AL, Capelo A, Dias CC. Laboratório de comunicação: ampliando as habilidades do estudante de medicina para a prática da entrevista. Interface (Botucatu). [online]. 2010. [capturado em: 25 mai. 2018]; 4(32): 217–227. Disponível em: http://www.scielo.br/scielo.php?pid=S1414-32832010000100018&script=sci_abstract&tlng=pt

[25] Sombra Neto LL, Silva VLL, Lima CDC, Moura HTM, Gonçalves ALM, Pires APB, Fernandes VG. Habilidade de comunicação da má notícia: o estudante de medicina está preparado? RevBrasEduc Med. [online]. 2017. [capturado em: 15 abr. 2018]; 41(2): 260–268. Disponível em: http://www.scielo.br/scielo.php?script=sci_arttext&pid=S0100−−55022017000200260&lng=en&nrm=iso&tlng=pt

[26] Bruner JS. The narrative construction of reality. CriticalInquiry. [online]. 1991. [capturado em: 29 mai. 2018]; 18(1): 1–21. Disponível em: https://philpapers.org/rec/BRUTNC

[27] Mello TB. Comunicação de más notícias: experiência de mães de crianças e adolescentes com câncer. [Dissertação Mestrado]. Ribeirão Preto: Escola de Enfermagem de Ribeirão Preto; 2013, 90p. [capturado em: 28 mai. 2018] Disponível em: http://www.teses.usp.br/teses/disponiveis/22/22133/tde-14012014-152912/pt-br.php

[28] Nakamoto PS. A humanização no Pronto Socorro do Hospital das Clínicas da Faculdade de Medicina de Botucatu sob a perspectiva dos profissionais de saúde. Interface (Botucatu). [online]. 2008. [capturado em: 7 abr. 2018]; 12(26): 684–685. Disponível em: https://repositorio.unesp.br/handle/11449/30406

[29] Sontag S A doença como metáfora. Rio de Janeiro: Edições Graal, 1984.

[30] Turato ER. Tratado da metodologia da pesquisa clínico--qualitativa: construção teórico-epistemológica, discussão comparada e aplicação nas áreas da saúde e humanas. Rio de Janeiro: Vozes, 2011.

